# Genome Sequences of Chikungunya Virus Strains from Bangladesh and Thailand

**DOI:** 10.1128/MRA.01452-19

**Published:** 2020-01-09

**Authors:** Alyssa T. Pyke, Jamie McMahon, Peter Burtonclay, Neelima Nair, Amanda De Jong

**Affiliations:** a Public Health Virology Laboratory, Forensic and Scientific Services, Coopers Plains, Queensland, Australia; DOE Joint Genome Institute

## Abstract

We sequenced the genomes of two chikungunya virus isolates obtained from viremic patients who had traveled to Australia. The first patient acquired the infection in Bangladesh in 2017, and the second was infected in Thailand in 2019. Phylogenetic sequence analysis demonstrated that both isolates belonged to the East/Central/South African genotype.

## ANNOUNCEMENT

Within the last 20 years, the reemergence and global spread of the arthropod-borne chikungunya virus (CHIKV) (genus *Alphavirus*, family *Togaviridae*) have resulted in explosive epidemics throughout the Pacific, Asia, and the Americas, affecting millions of people (1). Large-scale CHIKV outbreaks were recently recorded in Dhaka, Bangladesh, in 2017 (2) and Thailand in 2019 (3). Areas of nonendemicity, such as Queensland, Australia, which harbor populations of Aedes aegypti and Aedes albopictus mosquitoes, are at risk of CHIKV importation by viremic travelers and subsequent autochthonous transmission (1, 4).

In July 2017, a febrile male patient traveled to Brisbane, Australia, from Bangladesh. Similarly, in September 2019, a female patient with fever and joint pains arrived in Brisbane after recent travel in Thailand. Acute-phase sera were analyzed using a specific CHIKV reverse-transcription real-time PCR assay (4) which detected CHIKV RNA in each patient sample. The same patient sera were inoculated onto *A. albopictus* C6/36 cell monolayers. Two CHIKV isolates, namely, Bangladesh 2017 and Thail 2019, were recovered and subsequently used for whole-genome sequencing (WGS) and phylogenetic analysis.

To support ongoing and enhanced CHIKV surveillance within Australasia and provide contemporary sequences for continued scrutiny of existing molecular diagnostic assays, we performed WGS as previously described (5, 6). Briefly, total RNA was extracted from passage 1 C6/36 culture supernatants from each of the Bangladesh 2017 and Thail 2019 isolates using the QIAamp viral RNA extraction kit (Qiagen, Chadstone, Australia) without carrier RNA. Host and potentially contaminating microbial DNA was removed with DNase treatment (Heat&Run kit; ArcticZymes, Scientifix, South Yarra, Australia). RNA was converted to cDNA using the ProtoScript II first-strand cDNA kit (New England Biolabs), and second-strand cDNA synthesis was performed using an enzyme cocktail of Escherichia coli DNA ligase, DNA polymerase I, and RNase H (New England Biolabs). The Nextera XT kit was used for cDNA library construction, and paired-end (2 × 151 nucleotides [nt]) sequencing was performed using the V2 midoutput kit on a NextSeq 500 machine (Illumina, San Diego, CA).

Illumina sequencing yielded 10,299,664 and 16,336,098 reads for Bangladesh 2017 and Thail 2019, respectively, and raw sequence reads were processed using Geneious R10 version 10.2.6 software (7). Complete genome sequence assembly was achieved by mapping to a reference CHIKV genome sequence (strain CHIK31, GenBank accession number EU564335) that included 5′ and 3′ untranslated terminal sequences, using default parameters and the low-sensitivity setting. For Bangladesh 2017 (11,812 nt with 50.1% G+C content), a total of 4,908,657 reads with an average coverage depth of 45,504× were mapped to strain CHIK31. Similarly, a total of 8,240,564 reads with an average coverage depth of 78,217× were mapped to strain CHIK31 for Thail 2019 (11,812 nt with 50.1% G+C content).

**FIG 1:**
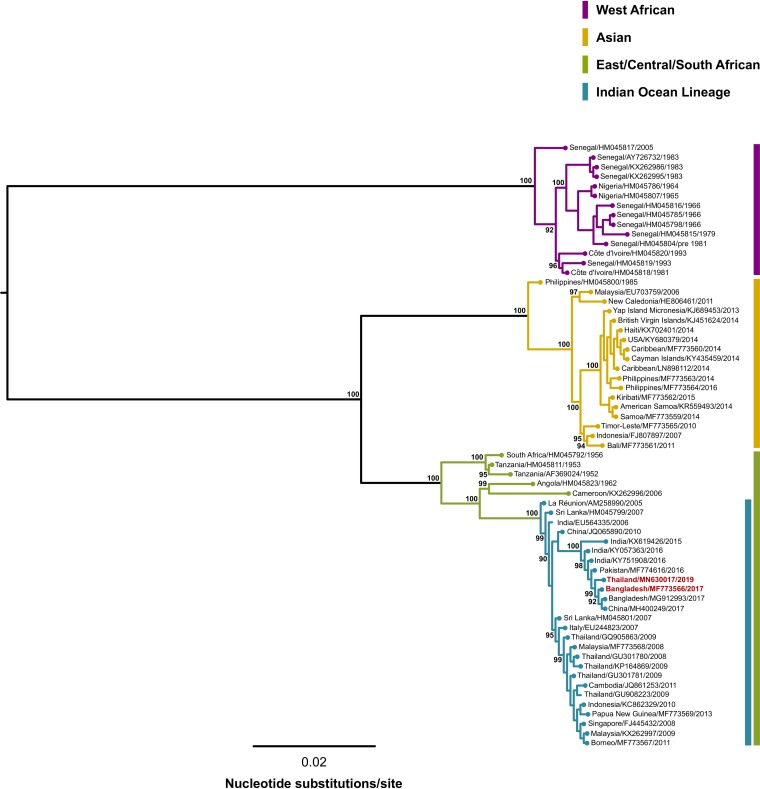
Approximately maximum-likelihood phylogenetic tree (midpoint rooted for branch visibility only) inferred for 63 CHIKV genome sequences (excluding 5′ and 3′ untranslated regions) using FastTree version 2.1.11 software and the GTR nucleotide substitution model with the default setting of 20 for rate categories of sites (8). Percentage Shimodaira-Hasegawa-like local support values are shown for key nodes. Multiple sequence alignments were performed using the Multiple Alignment using Fast Fourier Transform (MAFFT) program version 7.450 and Geneious version 10.2.6 software. The three major CHIKV genotypes (West African, Asian, and ECSA) are shown, including grouping of the Bangladesh 2017 (GenBank accession number MF773566) and Thail 2019 (GenBank accession number MN630017) isolates (red font) within the ECSA-Indian Ocean lineage.

Genome sequence identities were determined using the online version of blastn (https://blast.ncbi.nlm.nih.gov/Blast.cgi). Bangladesh 2017 and Thail 2019 shared 99.83% sequence identity with and were most closely related to a Chinese 2017 isolate, QZ0823 (GenBank accession number MH400249), which shared 99.95% and 99.85% sequence identity with Bangladesh 2017 and Thail 2019, respectively. Further sequence and phylogenetic analysis (FastTree software version 2.1.11 [8] and the general time-reversible [GTR] nucleotide substitution model) of the region encompassing the putative nonstructural polyprotein (nsP) (nsP1-nsP2-nsP3 termination product or nsP1-nsP2-nsP3-nsP4 read-through product), the subgenomic 26S RNA promoter region, and the putative structural polyprotein (C-E3-E2-6K-E1) indicated that both Bangladesh 2017 and Thail 2019 grouped within the Indian Ocean lineage of the East/Central/South African (ECSA) genotype (Fig. 1). However, the E1-A226V mutation first detected during the 2005–2006 CHIKV epidemic on La Réunion Island (9) was not found in either strain.

### Data availability.

Raw sequencing reads were deposited in the Sequence Read Archive under the accession numbers SAMN13164600 (Bangladesh 2017) and SAMN13164601 (Thail 2019) and BioProject number PRJNA583228. The genome sequences have been deposited in GenBank under the accession numbers MF773566 (Bangladesh 2017) and MN630017 (Thail 2019).
